# Supplementary material for: Acquisition of exogenous haem is essential for tick reproduction
Source: eLife. 2016 Mar 7;5:e12318. doi: 10.7554/eLife.12318 (PMC4821805; doi:10.7554/eLife.12318)
Supplement: Supplementary file 1. — DOI: http://dx.doi.org/10.7554/eLife.12318.023 [file elife-12318-supp1.docx]

| ***Ixodes scapularis* genes**  **coding for haemoproteins** | **No. of genes** | **Function** |
| --- | --- | --- |
| ***Cytochrome P450*** | **205** | **Detoxification / Lipid metabolism** |
| ***Cytochrome b5*** | **2** |  |
| ***Haem o synthase*** | **1** | **Haem synthesis** |
| ***Haem a synthase*** | **1** |  |
| ***Holocytochrome c synthase*** | **1** |  |
| ***Cytochrome b561*** | **2** | **Iron metabolism** |
| ***Ferric-chelate reductase*** | **1** |  |
| ***Succinate dehydrogenase cytochrome b*** | **1** | **Mitochondrial respiration** |
| ***Cytochrome b*** | **1** |  |
| ***Cytochrome c1*** | **1** |  |
| ***Cytochrome c*** | **2** |  |
| ***Cytochrome c oxidase I*** | **1** |  |
| ***Dual oxidase*** | **1** | **Redox homeostasis** |
| ***Catalase*** | **1** |  |
| ***Soluble guanylyl cyclase*** | **2** | **Nitric oxide metabolism** |
| ***Nitric oxid synthase*** | **1** |  |
| ***Tryptophan 2,3-dioxygenase*** | **1** | **Tryptophan metabolism** |

**Supplementary Table 2: Oligonucleotides used in this work**

| Amplicon name | Forward primer 5´- 3´ | Reverse primer 5´- 3´ | Amplicon length (bp) |
| --- | --- | --- | --- |
| CP3_qPCR | AGGCGAATCAAAGCGTAAGA | TGGGTACCATAACGCAGATG | 62 |
| Vg1_qPCR | GTACGACAACGTGAGCTAC | TGCAGTCTCCAGTAAGCAGGTCC | 68 |
| Vg2_qPCR | CCTCACTTCCCTCCAGACTTC | CCAGGAGCAAGTCGAGGAC | 62 |
| EF1_qPCR | ACGAGGCTCTGACGGAAG | CACGACGCAACTCCTTCAC | 81 |
| Actin_qPCR | CGACATCAAGGAGAAGCTCTG | GTCGGGAAGCTCGTAGGAC | 103 |
| CP3_pet100 | caccTTCGAAGTGGGCAAGGAC | tcactattaGCGGAAGGCGCTGGTCACG | 1819 |
| Vg1_pet100 | caccGTCTACAAGGTCAATGGTACC | ttattaCTTGAGGGCAGTGTACACGTA | 2080 |
| Vg2_pet100 | caccTTCGAGCCGAACCAGGAAT | ttattaCAGCGTAGAGTAGGTGAACG | 2161 |
| *Ir*Fer1_pet100 | caccATGGCCGCCACTCAGC | ctattaTCAGTCGGACAGGGTCTCC | 529 |
| CP3_RNAi | atgggcccCCTCGACCTAGAAAGGCAC | attctagaGTGCAGCTGGAACGACGGTG | 521 |
| Vg1_RNAi | atgggcccGTACAAGCACACGTACTACAA | attctagaCTTGAAAAGACTGGTCTCG | 317 |
| Vg2_RNAi | atgggcccGACCCACCTGAAGAACGAC | attctagaTACAGACTCAGGTGCTCGAG | 319 |
| *Ir*Fer1_RNAi | atggtaccAAACGGTTCGCTTTCCTC | attctaGAGCCCCACTCGTCCTGGG | 380 |
| IRP_RNAi | atgggccCAGCAAGAACTGGCAGAG | attctagaCAGGTGCAGGGTGCGTGG | 391 |

**Supplementary file 1: Design, sequences, and sequence similarities of recombinant Vitellogenin_N domains of *Ir*CP3, *Ir*Vg1, and *Ir*Vg2 used for raising specific antibodies**

| ***Ir*CP3 (GenBank KP663716)** |
| --- |
| **Domain structure**  **vWD**  (1356‒1514)  **DUF1943**  (658‒945)  **Vitellogenin_N**  (18‒619)    1  **-C**  **N-**  1537 |
| **His-tagged recombinant fragment - r*Ir*CP3 (18-619)**  MRGSHHHHHHGMASMTGGQQMGRDLYDDDDKDHPFTFEVGKDYVYHYNGKMQVYNPEQPLQSSGFAFRSKVVAQPRPDHTHFKIIDFEVDSFNGDHVHVGEHEFNYHSTEALKQFIERPFAGKFSEGKLEEAELSKSEPKWARNLKKGVLSIFQLDLVKGRHDHPHAKQFHVREEGLHGNCDTLYVVAEEEGHLKVTKIKNLEKCDKEHYAVYGRIKGHECVDCEAQETHPFVATSQVKYRLDGTPEHYVINHACATSENVFRPFGQGKTFVAQLNRTLDLEEVHDANTDTQLPEDLEKVHHIAQTFPESDEVESLEELKHVNRYVTTFDLSTDKDKFISGLNHLAALEYEDSDIKDVHSKESGGLNFLILFGSLASMPFEDIAHVYEQAVANAPEASKSQVRKVFLDLLSAVGNNPHAAFGLQLVKEDKLTDEEAEHFLAKLALNLKENSPALLTELAEVCEHVKPKRPVWVNCQLALSTLAGQEGCVRAKTDKEQDEGFCKPSIVSHFFNYEIKPEDKKDQPEYKRTVYMKAAGNLATRGAVHYLERYVSDTNQPEYRRSAALWAMVRAAPHHHELVRDVALPLYKNKSETAYLRIGAFVNVLMTKPDLYLLKYIGHNIIDDPSDQLASYVTSAFR |
| ***Is*Vg1 (ISCW013727)** |
| **Domain structure**  **Vitellogenin_N**  (33‒243,262‒723)  **DUF1943**  (755‒992)  **vWD**  (1491‒1659)  1  **-C**  **N-**  1936 |
| **His-tagged recombinant fragment - r*Ir*Vg1 (34‒723)**  MRGSHHHHHHGMASMTGGQQMGRDLYDDDDKDHPFTVYKVNGTVTLKTLELDVTEGPALTYEGDLAVQKLTETDYVAKFLNFTLVKFDKVLGDVHHFEPHYESSLYGQEVDYFQHLQYPVRFTLKQGKVVEYGVAQEVRAGALNVYKAVLTLLQSQPETFQELPTVVSYYEDGVSGYCRVNYELQSLDAHVYTGANVVNVTKTKYLDDCKKTRPVYTVDSVEVQGYPPLCNKHLPNNFLPGYQEDTAEYEASPTVGCPVGYKPFNTLVTAHEVSYYNLSDNVLESAYTESLDVLNVFTGKVVVKTLLKVLLAHVDGPQLEEFTPVQTYQTLELTLPETSHYFDLPVYSLLVETPEEGPLKFPEALTTVVDELVSLEVEDNTAEPKQTPGLLLQLVKTVGVLTFEQLKQTVPEFLQRPVLELAPHEQVHRSLWVDLVGKAGSKSSLDLVLYLLEQNLLTRNEARRVLQDVAAFKAYPEKETLEKYLEFALGQSQVLPPLVFSTLLHTLGELVNEACPSEVEYSSYEEGYLVEVEEHAPLHRLSLPVGAQCTVQDLQQYVLRISEALKQTDDFKKVVAYLHGLGKFAKPEVLPVLLAYVNGTAENLYRLVSEGEDYLESVYFVRKAALLALDHVVKYYPKEVSPLVRTLVLNTTEPTDLRTLAFDIWLKSVPAKWDLQQVVLAAKTDLSLEFGTYEGTALK |
| ***Is*Vg2 (ISCW021228)** |
| **Domain structure**  **Vitellogenin_N**  (28‒248,255‒745)  **VWD**  (1321‒1488)    1  **-C**  **N-**  1644 |
| **His-tagged recombinant fragment - r*Ir*Vg2(28‒744)**  MRGSHHHHHHGMASMTGGQQMGRDLYDDDDKDHPFTFEPNQEYLYKYRTAVSLSLPLKATHATGEETYGLLSVVVKEASGTGRSLVLQLLNVTSTLYDKEVEDQTEPVPGVYHQPLPVFESYQTGPVVLKLVDHSVESLEVPVGVPEEVVNLYRGLASVLTLSNPSYKKVPFTKEVPLALNDDVVVYKVYEDDLVGTCETVYNVLSSPHDEYVLNFTKTKNYHKCVGKTTVFQHVDYEHSGCPHACLKHQPKPLSETLEPELSDYVDPYGGGCPTETHLKNDLAESFLTVHYNVSLHQEVGVLEEVKAIDKKVLTSGKQQLVSTSVLHLELLLKTTPFTAVGPLEDVKTYTNLSYVYPKQHYSWHGQLYELEHLSLYGPVDTVEARTAVRGLLDQLAGLLVLDDLEVKDDYADLVSQLLTAVNVLKEYDLELLLQTVVPLENVKVVSEKEYIERKLLLDVLSLAGTDAAAKTVLRLLLEQKLTLVEAVHVLTSLQTSLVKPSTEVLDLLLDLATGGVLEKDRLLYSTAYLTLAKVVSKHCHLYDTTSHVPYGKLLRMNELDAIKKKTVPHLPTYRSMKTLKPRMSGRQYQETETEEPQYTGVPVTCTSQDYLKYVQALVQKLNEAKEFHQVTVLVHALTQLQHPEALKALVPVVLGKHHLCQTTLPEEEQSESCQYLRLVTLYALRHSLKHHAAEIQPLAQTVYFNTDEDYELRNAALVLLMGSHPPEPVLARVVLTLQKELNLQVASFTTLRCN |
| **Amino-acids identity matrix of recombinant fragments**   \| **r*Ir*CP3** \| **r*Ir*Vg1** \| **r*Ir*Vg2** \|  \| \| --- \| --- \| --- \| --- \| \|  \| **14.5%** \| **15.8%** \| **r*Ir*CP3** \| \|  \|  \| **22.3%** \| **r*Ir*Vg1** \| \|  \|  \|  \| **r*Ir*Vg2** \| |
