# Supplementary material for: Acquisition of exogenous haem is essential for tick reproduction
Source: eLife. 2016 Mar 7;5:e12318. doi: 10.7554/eLife.12318 (PMC4821805; doi:10.7554/eLife.12318)
Supplement: Supplementary file 2. — DOI: http://dx.doi.org/10.7554/eLife.12318.024 [file elife-12318-supp2.docx]

| ***Ir*CP3 (GenBank KP663716)** |
| --- |
| **Domain structure**  **vWD**  (1356‒1514)  **DUF1943**  (658‒945)  **Vitellogenin_N**  (18‒619)    1  **-C**  **N-**  1537 |
| **His-tagged recombinant fragment - r*Ir*CP3 (18-619)**  MRGSHHHHHHGMASMTGGQQMGRDLYDDDDKDHPFTFEVGKDYVYHYNGKMQVYNPEQPLQSSGFAFRSKVVAQPRPDHTHFKIIDFEVDSFNGDHVHVGEHEFNYHSTEALKQFIERPFAGKFSEGKLEEAELSKSEPKWARNLKKGVLSIFQLDLVKGRHDHPHAKQFHVREEGLHGNCDTLYVVAEEEGHLKVTKIKNLEKCDKEHYAVYGRIKGHECVDCEAQETHPFVATSQVKYRLDGTPEHYVINHACATSENVFRPFGQGKTFVAQLNRTLDLEEVHDANTDTQLPEDLEKVHHIAQTFPESDEVESLEELKHVNRYVTTFDLSTDKDKFISGLNHLAALEYEDSDIKDVHSKESGGLNFLILFGSLASMPFEDIAHVYEQAVANAPEASKSQVRKVFLDLLSAVGNNPHAAFGLQLVKEDKLTDEEAEHFLAKLALNLKENSPALLTELAEVCEHVKPKRPVWVNCQLALSTLAGQEGCVRAKTDKEQDEGFCKPSIVSHFFNYEIKPEDKKDQPEYKRTVYMKAAGNLATRGAVHYLERYVSDTNQPEYRRSAALWAMVRAAPHHHELVRDVALPLYKNKSETAYLRIGAFVNVLMTKPDLYLLKYIGHNIIDDPSDQLASYVTSAFR |
| ***Is*Vg1 (ISCW013727)** |
| **Domain structure**  **Vitellogenin_N**  (33‒243,262‒723)  **DUF1943**  (755‒992)  **vWD**  (1491‒1659)  1  **-C**  **N-**  1936 |
| **His-tagged recombinant fragment - r*Ir*Vg1 (34‒723)**  MRGSHHHHHHGMASMTGGQQMGRDLYDDDDKDHPFTVYKVNGTVTLKTLELDVTEGPALTYEGDLAVQKLTETDYVAKFLNFTLVKFDKVLGDVHHFEPHYESSLYGQEVDYFQHLQYPVRFTLKQGKVVEYGVAQEVRAGALNVYKAVLTLLQSQPETFQELPTVVSYYEDGVSGYCRVNYELQSLDAHVYTGANVVNVTKTKYLDDCKKTRPVYTVDSVEVQGYPPLCNKHLPNNFLPGYQEDTAEYEASPTVGCPVGYKPFNTLVTAHEVSYYNLSDNVLESAYTESLDVLNVFTGKVVVKTLLKVLLAHVDGPQLEEFTPVQTYQTLELTLPETSHYFDLPVYSLLVETPEEGPLKFPEALTTVVDELVSLEVEDNTAEPKQTPGLLLQLVKTVGVLTFEQLKQTVPEFLQRPVLELAPHEQVHRSLWVDLVGKAGSKSSLDLVLYLLEQNLLTRNEARRVLQDVAAFKAYPEKETLEKYLEFALGQSQVLPPLVFSTLLHTLGELVNEACPSEVEYSSYEEGYLVEVEEHAPLHRLSLPVGAQCTVQDLQQYVLRISEALKQTDDFKKVVAYLHGLGKFAKPEVLPVLLAYVNGTAENLYRLVSEGEDYLESVYFVRKAALLALDHVVKYYPKEVSPLVRTLVLNTTEPTDLRTLAFDIWLKSVPAKWDLQQVVLAAKTDLSLEFGTYEGTALK |
| ***Is*Vg2 (ISCW021228)** |
| **Domain structure**  **Vitellogenin_N**  (28‒248,255‒745)  **VWD**  (1321‒1488)    1  **-C**  **N-**  1644 |
| **His-tagged recombinant fragment - r*Ir*Vg2(28‒744)**  MRGSHHHHHHGMASMTGGQQMGRDLYDDDDKDHPFTFEPNQEYLYKYRTAVSLSLPLKATHATGEETYGLLSVVVKEASGTGRSLVLQLLNVTSTLYDKEVEDQTEPVPGVYHQPLPVFESYQTGPVVLKLVDHSVESLEVPVGVPEEVVNLYRGLASVLTLSNPSYKKVPFTKEVPLALNDDVVVYKVYEDDLVGTCETVYNVLSSPHDEYVLNFTKTKNYHKCVGKTTVFQHVDYEHSGCPHACLKHQPKPLSETLEPELSDYVDPYGGGCPTETHLKNDLAESFLTVHYNVSLHQEVGVLEEVKAIDKKVLTSGKQQLVSTSVLHLELLLKTTPFTAVGPLEDVKTYTNLSYVYPKQHYSWHGQLYELEHLSLYGPVDTVEARTAVRGLLDQLAGLLVLDDLEVKDDYADLVSQLLTAVNVLKEYDLELLLQTVVPLENVKVVSEKEYIERKLLLDVLSLAGTDAAAKTVLRLLLEQKLTLVEAVHVLTSLQTSLVKPSTEVLDLLLDLATGGVLEKDRLLYSTAYLTLAKVVSKHCHLYDTTSHVPYGKLLRMNELDAIKKKTVPHLPTYRSMKTLKPRMSGRQYQETETEEPQYTGVPVTCTSQDYLKYVQALVQKLNEAKEFHQVTVLVHALTQLQHPEALKALVPVVLGKHHLCQTTLPEEEQSESCQYLRLVTLYALRHSLKHHAAEIQPLAQTVYFNTDEDYELRNAALVLLMGSHPPEPVLARVVLTLQKELNLQVASFTTLRCN |
| **Amino-acids identity matrix of recombinant fragments**   \| **r*Ir*CP3** \| **r*Ir*Vg1** \| **r*Ir*Vg2** \|  \| \| --- \| --- \| --- \| --- \| \|  \| **14.5%** \| **15.8%** \| **r*Ir*CP3** \| \|  \|  \| **22.3%** \| **r*Ir*Vg1** \| \|  \|  \|  \| **r*Ir*Vg2** \| |
