# Supplementary material for: Acquisition of exogenous haem is essential for tick reproduction
Source: eLife. 2016 Mar 7;5:e12318. doi: 10.7554/eLife.12318 (PMC4821805; doi:10.7554/eLife.12318)
Supplement: Supplementary file 3. — DOI: http://dx.doi.org/10.7554/eLife.12318.025 [file elife-12318-supp3.docx]

| Amplicon name | Forward primer 5´- 3´ | Reverse primer 5´- 3´ | Amplicon length (bp) |
| --- | --- | --- | --- |
| CP3_qPCR | AGGCGAATCAAAGCGTAAGA | TGGGTACCATAACGCAGATG | 62 |
| Vg1_qPCR | GTACGACAACGTGAGCTAC | TGCAGTCTCCAGTAAGCAGGTCC | 68 |
| Vg2_qPCR | CCTCACTTCCCTCCAGACTTC | CCAGGAGCAAGTCGAGGAC | 62 |
| EF1_qPCR | ACGAGGCTCTGACGGAAG | CACGACGCAACTCCTTCAC | 81 |
| Actin_qPCR | CGACATCAAGGAGAAGCTCTG | GTCGGGAAGCTCGTAGGAC | 103 |
| CP3_pet100 | caccTTCGAAGTGGGCAAGGAC | tcactattaGCGGAAGGCGCTGGTCACG | 1819 |
| Vg1_pet100 | caccGTCTACAAGGTCAATGGTACC | ttattaCTTGAGGGCAGTGTACACGTA | 2080 |
| Vg2_pet100 | caccTTCGAGCCGAACCAGGAAT | ttattaCAGCGTAGAGTAGGTGAACG | 2161 |
| *Ir*Fer1_pet100 | caccATGGCCGCCACTCAGC | ctattaTCAGTCGGACAGGGTCTCC | 529 |
| CP3_RNAi | atgggcccCCTCGACCTAGAAAGGCAC | attctagaGTGCAGCTGGAACGACGGTG | 521 |
| Vg1_RNAi | atgggcccGTACAAGCACACGTACTACAA | attctagaCTTGAAAAGACTGGTCTCG | 317 |
| Vg2_RNAi | atgggcccGACCCACCTGAAGAACGAC | attctagaTACAGACTCAGGTGCTCGAG | 319 |
| *Ir*Fer1_RNAi | atggtaccAAACGGTTCGCTTTCCTC | attctaGAGCCCCACTCGTCCTGGG | 380 |
| IRP_RNAi | atgggccCAGCAAGAACTGGCAGAG | attctagaCAGGTGCAGGGTGCGTGG | 391 |
